# Letter to the editor in response to a letter by Deeks regarding Peto T; UK COVID-19 lateral flow oversight team: COVID-19: Rapid antigen detection for SARS-CoV-2 by lateral flow assay

**DOI:** 10.1016/j.eclinm.2021.101043

**Published:** 2021-07-23

**Authors:** Lennard Y.W. Lee, Tim E. Peto

**Affiliations:** a Dept of Oncology, University of Oxford, England; b Nuffield Dept of Medicine, University of Oxford, Oxford OX3 9DU, England

We have read the letter from Jon Deeks with care. We are sorry that the writers are unhappy with the design of the School and Armed Forces studies (Phase 4 studies). The main evaluation of the lateral flow devices is reported in the Phase 3 studies. The Phase 4 studies were designed to determine the feasibility of using lateral flow devices in different settings. As the disease prevalence at the time of these studies was very low, the Phase 4 studies were not powered to undertake a standard prospective evaluation of both positive and negative cases.

We are happy to confirm that all the samples shown in Table 2 had both a negative PCR test and a lateral flow test allowing the false positive rate to be estimated. PHE Porton tested the PCR samples collected from the school and armed forces studies. We agree that for the Phase 2 and 3 studies, lateral flow tests were done in laboratory conditions but overall 5942/6954 (85.4%) of the tests were done following the manufacturing instructions (rather than the 1852 suggested by the writers).

The writers refer to a personal communication providing Prof Deeks preliminary with the results then available. The current publication provides a cleaned version of the results. The specificity results from the very small Armed Forces study was included as we were interested in the false positive rate, at a time of lower disease prevalence. We apologize for not including in this paper the sensitivity results which we sent directly to Prof Deeks for the Cochrane Review. These show that the numbers (pos/total) were 8/8 (CT < 25); 3/4 (CT > = 25 to 28) and 2/34 (CT > = 28). As the disease was waning in the population at the time, the prevalence of low viral loads is not surprising and the low positive rate in such a population is expected and fits with the more complete Phase 3a results presented in our paper.

We do not consider that an overall ‘sensitivity’ of the test against a categorical PCR result, without accounting for viral load, is a useful measure of LFD performance as all studies confirm that the LFD test is normally negative in individuals with low viral loads. The author's quoting of a sensitivity of only 28% and a positive predictive value of 18% at low disease prevalence is misleading, as already discussed as it assumes that the PCR test is a reliable test for infectiousness.

Since this study was undertaken, lateral flow tests have been widely used in the UK and elsewhere. Many thousands of infectious individuals have been identified and subsequently isolated successfully, breaking the chain of transmission. For many, the return to a semblance of normal life has been facilitated by lateral flow tests.  There is no practical alternative as the PCR test has poor specificity in identifying infectious cases, is inaccessible, expensive and has a slow turn-around rate.

## Declaration of Competing Interest

The authors do not have any conflicts of Interest

